# Ultra-performance liquid chromatography-quadrupole/time-of-flight mass spectrometry analysis of the impact of processing on toxic components of Kansui Radix

**DOI:** 10.1186/s12906-016-1039-7

**Published:** 2016-02-24

**Authors:** Xin Shu, Xi-Wen Jiang, Brian Chi-Yan Cheng, Shuang-Cheng Ma, Guang-Ying Chen, Zhi-Ling Yu

**Affiliations:** Consun Chinese Medicines Research Centre for Renal Diseases, School of Chinese Medicine, Hong Kong Baptist University, Kowloon Tong, Hong Kong; National Institutes for Food and Drug Control, Beijing, China; College of chemistry & chemical engineering, Hainan Normal University, Haikou, China

**Keywords:** Ultra-performance liquid chromatography-quadrupole/time-of-flight mass spectrometry, Kansui Radix, Processing with vinegar, Chemical profile, Toxic components

## Abstract

**Background:**

Kansui Radix (*Gansui* in Chinese), the dried tuber of *Euphorbia kansui*, is a Chinese medicinal herb commonly used for the treatment of oedema and ascites with dyspnea. Because of its toxic nature, the herb is usually processed with vinegar to reduce the toxicity. A report has shown that the contents of toxic terpenoids in *Gansui* decreased after processing with vinegar. However, comprehensive comparison of the chemical profiles between vinegar-processed and raw *Gansui* has not yet been conducted.

**Methods:**

An ultra-high-performance liquid chromatography in conjunction with ultra-high resolution quadrupole time-of-flight mass spectrometry (UHPLC UHD Q-TOF MS/MS) method was developed for the analysis of chemical profiles of vinegar-processed and raw *Gansui* in this study.

**Results:**

Results showed that processing with vinegar caused conspicuous chemical changes. Among the altered components, 11 toxic terpenoids, 3-*O*-benzoyl-13-*O*- dodecanoylingenol/20-*O*-benzoyl-13-*O*-dodecanoylingenol, kansuinine D, kansuinine A, 3-*O*-benzoyl-13-*O*-dodecanoylingenol/20-*O*-benzoyl-13-*O*-dodecanoylingenol, 3-*O*- benzoylingenol/20-*O*-benzoylingenol, 20-*O*-(*2*′*E*,*4*′*Z*-decadienoyl)ingenol/20-*O*-(*2′E*,*4′E*- decadienoyl)ingenol/3-*O*-(*2′E*,*4′Z*-decadienoyl)ingenol/3-*O*-(*2*′*E*,*4*′*E*-decadienoyl)ingenol, 3-*O*-*(2′E*,*4′Z*-decadienoyl)-20-deoxyingenol,3-*O*-(*2*′*E*,*4*′*Z*-,ecadienoyl)-5-*O*-acetylingenol,3-*O*-(*2*′*E*,*4*′*Z*-decadienoyl)-20-*O*-acetylingenol,3-*O*-(2,3-dimethylbutanoyl)-13-*O*-dodecanoylingenol, were tentatively identified. The contents of most of these terpenoids were obviously decreased after processing with reductions of 6.66–95.25 %.

**Conclusion:**

Our findings could help us understand the chemical basis for the toxicity reduction of *Gansui* afforded by processing with vinegar. Further investigations are warranted to establish the relationship between processing-induced chemical changes and the reduction of toxicity of *Gansui*.

## Background

Kansui Radix (*Gansui* in Chinese), the dried tuber of *Euphorbia kansui* T.N. Liou ex T.P. Wang., is a well-known traditional Chinese medicinal herb. It was first recorded in Shennong’s Compendium of Materia Medica (*Shennong Bencao Jing)* issued 2000 years ago. Studies have revealed *Gansui*’s antitumor [1–5], antivirus [6], antileukemia [4, 7], antiallergic [8] and nerve growth factor-promoting activities [9]. However, it also exhibited skin-irritating, tumor-promoting and inflammatory activities [6, 10]. Among the chemical constituents including diterpenoids, triterpenoids and phenolic derivatives [2, 3, 6, 9, 11] that had been identified in Gansui, ingenol type diterpenoids were proposed to be the main toxic components [12]. In order to reduce the toxicity, *Gansui* is usually processed with vinegar [13]. The content of six toxic terpenoids in *Gansui* and their hepatotoxicity were found to be decreased after processing with vinegar in a recent report [14]. However, the processing-induced chemical changes are still not fully understood. In the present study, a comprehensive comparison of the chemical profiles between vinegar-processed and raw *Gansui* was conducted. Eleven toxic compounds, which were reduced after processing, were tentatively identified using an established UHPLC UHD Q-TOF MS/MS method.

## Method

### Herbal materials

*Gansui,* the dried tuber of *E. kansui*. was purchased from Zisun Group (Hong Kong) Limited, and morphologically authenticated by Professor Zhi-Ling Yu (School of Chinese Medicine, Hong Kong Baptist University). A voucher specimen (Gansui-01) has been deposited at the School of Chinese Medicine, Hong Kong Baptist University.

Vinegar-processed *Gansui* was prepared by the following procedures: 10 g of cleaned herbal material was sliced (3–4 mm in thickness) and mixed evenly with 3 g vinegar in a beaker. The beaker was sealed until the vinegar was fully absorbed. After that, the herbal material was stir baked at a high temperature until the color deepened and scorched spots appeared slightly. The vinegar-processed *Gansui* and its corresponding raw *Gansui* were dried in a drying oven at 37 °C overnight.

After cooling down, both vinegar-processed and raw *Gansui* were crushed into powder and passed through a 24-mesh sieve. Each of the herbal materials (5 g, accurately weighed) were extracted 3 times with 50 ml 95 %, 70 %, 30 % ethanol separately by sonication for 1.5 h. The extracts were centrifuged at × 1800 g for 10 min. The supernatants were combined as the extraction solution. All extraction solutions were filtered through a 0.45 μm PTFE filter prior to UHPLC UHD Q-TOF MS/MS.

### Chemicals

Acetonitrile was of LC/MS grade (Fisher Scientific, Pittsburgh, PA, USA) and formic acid was of HPLC grade (Sigma-Aldrich, St. Louis, MO, USA). Ultra-pure water was prepared using a Milli-Q Plus water purification system (Millipore, Billerica, MA, USA). All other reagents used for extraction was of analytical grade. The reference compound kansuiphorin D was provided by Prof. Shuangcheng Ma (National Institutes for Food and Drug Control,Beijing,China), its retention time (t_R_) was 10.07 min under the UHPLC conditions described in the next section. Vinegar which complies with the standards of the Chinese Pharmacopoeia was obtained from the Pat Chun International Limited.

### Equipment and chromatographic conditions

The UHPLC conditions for LC-MS analysis were as follows: chromatography was performed on an Acquity UPLC T3 C18 column, 2.1 × 100 mm i.d., 1.8 μm (Waters Corp., Milford, MA, USA). The column was maintained at 40 °C. A gradient elution of solvent A (0.1 % formic acid in Milli-Q water) and solvent B (0.1 % formic acid acetonitrile) was applied as follows: 0–10 min, 50–75 % B; 10–15 min, 75–85 % B; 15–25 min, 85–95 % B; 25–30 min, 95–99 % B; 30–35 min, 99 % B; 35–40 min, 99–100 % B; 40–45 min, 100 % B. An equilibration period of 4.0 min was used between individual runs. The flow rate was 0.4 mL/min with 1 μL injection volume.

### Mass spectrometry conditions

An Agilent 6540 UHD Accurate-Mass Q-TOF mass spectrometer (Agilent Technologies, Santa Clara, CA, USA) was connected to the Agilent 1290 Infinity UHPLC system *via* an electrospray ionization (ESI) ion source with Jet-Stream technology for the comprehensive LC/MS/MS analysis of *Gansui* samples. The ESI-MS spectra in both positive and negative modes were acquired. Ultra-pure nitrogen (N_2_) was used as the nebulizing and sheath gas. Ultra-high-purity N_2_ was used as collision gas in product ion scanning experiments. The ESI parameters were set as follows: the capillary voltage was 4.5 kV. The flow rate and temperature of sheath gas were 8 L/min and 350 °C respectively. The flow rate and temperature of drying gas were 8 L/min and 300 °C, respectively. The pressure of nebulizer gas was 40 psi. The fragmentor voltage was 175 V. The mass analyzer was scanning from 100 to 1700 (*m/z)*. The Q-TOF acquisition rate was 2 Hz. The energies for collision-induced dissociation (CID) experiments were set at 10, 20 and 30 eV respectively.

### Data acquisition

The Q-TOF mass spectrometer was tuned in the low mass range (from 100 to 1700 Da) and in the extended dynamic range mode (2 GHz). All MS data were acquired with reference masses at *m/z* 112.9856 and 966.0007 in the negative ESI mode, and at *m/z* 121.0509 and 922.0098 in the positive ESI mode to ensure the mass accuracy and reproducibility.

## Results and discussion

The established ultra-high-performance liquid chromatography in conjunction with ultra-high resolution quadrupole time-of-flight mass spectrometry (UHPLC UHD Q-TOF MS/MS) was a quick and versatile method for comprehensive analysis of the chemical profiles of vinegar-processed and raw *Gansui* samples. The UHPLC coupled with sub-2-micron liquid chromatography provided strategies to improve resolution while maintaining or even shortening the overall running time. Q-TOF MS allowed accurate automated mass measurement of product ions for structural analysis. The prominent chromatographic resolution of MS and MS/MS was provided by the exact mass measurement of UHPLC UHD Q-TOF MS/MS.

### Comparison of the chemical profiles of vinegar-processed and raw Gansui samples

**Fig. 1:**
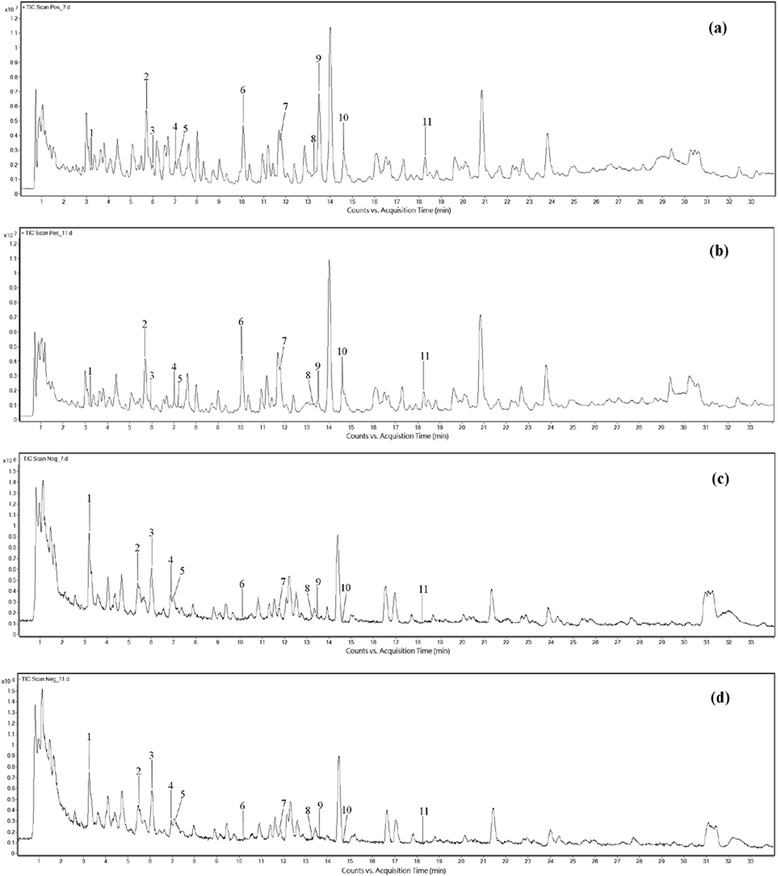
Representative total ion current chromatograms of *Gansui* samples. **a** Raw *Gansui* in the positive-ion mode; **b** Vinegar-processed *Gansui* in the positive-ion mode; **c** Raw *Gansui* in the negative-ion mode; **d** Vinegar-processed *Gansui* in the negative-ion mode

To compare the vinegar-processed and raw *Gansui* samples, global chemical profiling was conducted by UHPLC UHD Q-TOF MS in positive and negative ion modes. The representative total ion current (TIC) chromatograms of both samples are shown in Fig. 1. The changes were more remarkable in the positive-ion mode (Fig. 1a, b) in comparison with the negative-ion mode (Fig. 1c, d). As shown in Fig. 1a, b, the intensities of several peaks decreased significantly after processing with vinegar, suggesting a processing-induced chemical change in *Gansui*.

### Identity elucidation of detectable components in vinegar-processed and raw Gansui samples

**Fig. 2:**
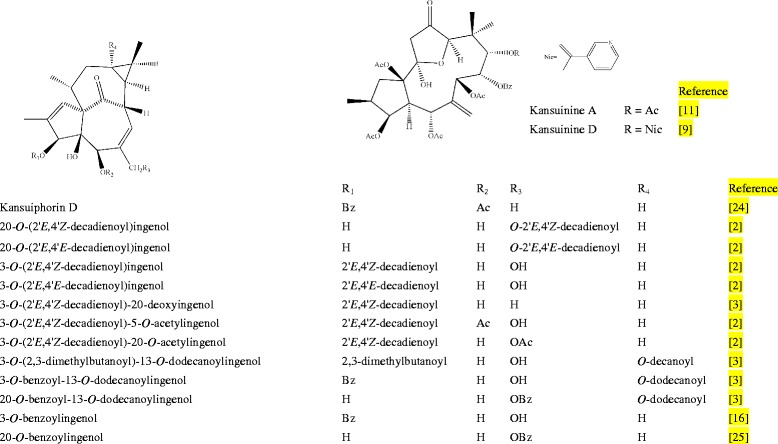
The chemical structures of diterpenoids identified in *Gansui* samples. [2, 3, 9, 11, 16, 24, 25]

Among the changed components, 11 peaks were tentatively identified. Their chemical structures were shown in Fig. 2. One of them was confirmed by comparing the mass spectra and retention time with those of the reference compounds, and the others were tentatively assigned by matching the empirical molecular formula deduced by matching mass values of quasi-molecular ions and fragment ions with its theoretical values and those of the known compounds in the literature.

Peak 1 showed a [M-H]^-^ ion at *m/z* 649.1583 in the negative-ion mode and a [M + Na]^+^ ion at *m/z* 673.2734 in the positive-ion mode. The ion at *m/z* 673.2734 could be further fragmented into ions at *m/z* 473.3624 [M + Na-C_12_H_24_O_2_]^+^ and 351.2374 [M + Na-C_12_H_24_O_2_-C_7_H_6_O_2_]^+^. These fragmented ions corresponded to the loss of a long chain and a benzoyloxy group. It was tentatively identified as 3-*O*-benzoyl-13-*O*-dodecanoylingeno or 20-*O*-benzoyl-13- *O*-dodecanoylingenol according to the MS data in the literature [15, 16]. These compounds are the skin-irritative ingenol-type diterpenoids in *Gansui* [17]. However, to unambiguously identify the isomeric compounds, other spectroscopic methods such as NMR were needed.

Peak 2 was tentatively identified as kansuinine D, which is a skin-irritative [17] and inflammatory [15] diterpenoid. It displayed a [M-H]^-^ ion at *m/z* 792.2738 in the negative-ion mode, a precursor ion at *m/z* 816.2931 [M + Na]^+^ and a fragment ion at *m/z* 756.2880 [M-C_2_H_4_O_2_ + Na]^+^ in the positive-ion mode, suggesting the presence of an acetoxy group. Several ions with low mass to charge ratio (at *m/z* 369.3048, 327.1625, 309.1534 and 281.2695) in the positive-ion mode were observed, which might be the fragments of the main skeleton. The MS data were in agreement with those in the literature [15, 16].

Peak 3 gave a [M-H]^-^ ion at *m/z* 729.2786 in the negative-ion mode and a [M + Na]^+^ ion at *m/z* 753.2812 in the positive-ion mode. In the MS/MS spectrum, the ions at *m/z* 633.5187 [M + Na-2C_2_H_4_O_2_]^+^, 631.5071 [M + Na-C_7_H_6_O_2_]^+^ and 453.3395 [M + Na-5C_2_H_4_O_2_]^+^ were observed, indicating the existence of five acetoxys and a benzoyloxy groups. The ions at *m/z* 369.3039, 341.3193 and 281.2689 might be the fragments of the main skeleton. Comparing with the literature [15, 16], peak 3 was characterized tentatively as kansuinine A. It has inflammatory activity [15] and cytotoxicity against human normal liver cell line L-O2 and gastric epithelial cell line GES-1 [18].

Peak 4 showed a [M-H]^-^ ion at *m/z* 649.3470 in the negative-ion mode and a [M + Na]^+^ ion at *m/z* 673.3103 in the positive-ion mode. In the MS/MS spectrum, the ions at *m/z* 473.3667 [M + Na-C_12_H_24_O_2_]^+^ and 351.1501 [M + Na-C_12_H_24_O_2_-C_7_H_6_O_2_]^+^ were observed, indicating the existence of a long-chain group and a benzoyloxy group. Comparing with the literature [15, 16], peak 4 was characterized tentatively as 3-*O*-benzoyl-13-*O*-dodecanoylingenol or 20-*O*-benzoyl-13-*O*-dodecanoylingenol. They are skin-irritative diterpenoids in *Gansui* [17].

Peak 5 gave an ion at *m/z* 433.2364 [M-H-H_2_O]^-^ in the negative-ion mode and a precursor ion at *m/z* 475.3825 [M + Na]^+^ in the positive-ion mode. The fragment ions at *m/z* 457.3708 [M + Na-H_2_O]^+^ and 353.1428 [M + Na-C_7_H_6_O_2_]^+^ in the positive-ion mode were observed, suggesting the existence of a benzoyloxy group, therefore it was tentatively characterized as 3-*O*-benzoylingenol or 20-*O*-benzoylingenol in accordance with the published data [15, 16]. 3-*O*-benzoylingenol is an aversion-inducing compound [19] with tumor promotion and skin-irritating activities [4]. However, to unambiguously identify the isomeric compounds, other spectroscopic method such as NMR was needed.

Peak 6 was confirmed as kansuiphorin D by comparing the mass spectra and the retention time (t_R_ = 10.07 min) with those of the reference compound, which is a tumor promoting, skin-irritating [4] compound isolated from *Gansui*. As there was almost no change in the intensity of this peak in raw and processed *Gansui* samples, it would not be discussed in detail.

For peak 7, a sodium adduct molecular ion at *m/z* 521.2948 [M + Na]^+^ in the negative-ion mode, and a fragment ion at *m/z* 497.2814 [M-H]^-^ in the positive-ion mode were observed. In MS/MS spectrum, the ions at *m/z* 503.2292 [M + Na-H_2_O]^+^, 353.1763 [M + Na-C_10_H_16_O_2_]^+^ and 335.1564 [M + Na-C_10_H_16_O_2_-H_2_O]^+^ were observed, suggesting the presence of a long chain group. According to the MS data of published data [15, 16], it was tentatively identified as 20-*O*-(*2′E,4′Z*-decadienoyl) ingenol or 20-*O*-(*2′E,4′E*-decadienoyl) ingenol or 3-*O*-(*2′E,4′Z*-decadienoyl) ingenol or 3-*O*-(*2′E,4′E*-decadienoyl) ingenol. 3-*O*-(*2′E,4′Z*-decadienoyl) ingenol, 3-*O*-(*2′E,4′E*-decadienoyl) ingenol and 20-*O*-(*2′E,4′Z*-decadienoyl) ingenol are skin-irritating ingenols, and 3-*O*-(*2′E,4′E*-decadienoyl) ingenol has tumor promotion activity [4, 17]. 20-*O*-(*2′E,4′E*-decadienoyl) ingenol has inflammatory activity [15].

Peak 8 showed a [M-H]^-^ ion at *m/z* 481.2945 in the negative-ion mode, a [M + Na]^+^ ion at *m/z* 505.2980 in the positive-ion mode and a fragment ion at *m/z* 337.1818 [M + Na-C_10_H_16_O_2_]^+^ by the loss of a long chain group. In agreement with reported data [15, 16], therefore, peak 9 was tentatively characterized as 3-O-(*2′E*,*4′Z*-decadienoyl)-20-deoxyingenol. It has inflammatory activity [15] and cytotoxicity against human normal liver cell line L-O2 [18].

Peaks 9, as shown in Table 1, gave the [M + HCOO]^-^ ion at *m/z* 585.3019 in negative -ion mode and a [M + Na]^+^ ion at *m/z* 563.5580 in the positive-ion mode. In the MS/MS spectrum, the ions at *m/z* 503.2812 [M + Na-C_2_H_4_O_2_]^+^ and 395.1912 [M + Na-C_10_H_16_O_2_]^+^ were observed, suggesting the presence of an acetoxy and a long chain groups. For peak 10, it gave the [M-H]^-^ ion at *m/z* 539.0378 in the negative-ion mode and the [M + Na]^+^ ion at *m/z* 563.3061 in the positive-ion mode. In the MS/MS spectrum, the ions at *m/z* 503.3406 [M + Na-C_2_H_4_O_2_]^+^, 395.1876 [M + Na-C_10_H_16_O_2_]^+^ and 335.1636 [M + Na-C_10_H_16_O_2_- C_2_H_4_O_2_]^+^ were observed, suggesting the existence of an acetoxy and a long chain groups. Therefore peaks 9 and 10 were tentatively identified as 3-*O*-(*2′E,4′Z-*decadienoyl)- 5-*O*-acetylingenol and 3-*O*-(*2′E,4′Z*-decadienoyl)-20-*O*-acetylingenol, respectively, which were further confirmed by comparing the retention time and MS data with published data in the literature [15, 16, 20, 21]. 3-*O*-(*2′E,4′Z-*decadienoyl)-5-*O*-acetylingenol has inflammatory activity [14, 15] and 3-*O*-(*2′E,4′Z*-decadienoyl)-20-*O*-acetylingenol is a skin-irritating component in *Gansui* [17].

**Table 1:** Diterpenoids identified in vinegar-processed and raw *Gansui* samples

Peak no.	RT (min)	*m/z* (ESI^+^) [M + Na]^+^	*m/z* (ESI^-^)	Identification	Decreased rate %
1	3.24	673.2734 473.3624 351.2374	649.1583 [M-H]^-^	3-*O*-benzoyl-13-*O*-dodecanoylingenol/ 20-*O*-benzoyl-13-*O*-dodecanoylingenol	32.16
2	5.66	816.2931 756.2880 369.3048 327.1625 309.1534 281.2695	792.2738 [M-H]^-^	Kansuinine D	22.21
3	5.99	753.2812 633.5187 631.5071 453.3395 369.3039 341.3193 281.2689	729.2786 [M-H]^-^	Kansuinine A	15.23
4	7.02	673.3103 473.3667 351.1501	649.3470 [M-H]^-^	3-*O*-benzoyl-13-*O*-dodecanoylingenol/ 20-*O*-benzoyl-13-*O*-dodecanoylingenol	24.85
5	7.18	475.3825 457.3708 353.1428	433.2364 [M-H_2_O-H]^-^	3-*O*-benzoylingenol/20-*O*-benzoylingenol	7.89
7	11.80	521.2948 503.2292 353.1763 335.1564	497.2814 [M-H]^-^	20-*O*-(*2*′*E*,*4*′*Z*-decadienoyl)ingenol/ 20-*O*-(*2′E*,*4′E*-decadienoyl)ingenol/ 3-*O*-(*2′E*,*4′Z*-decadienoyl)ingenol/ 3-*O*-(*2*′*E*,*4*′*E*-decadienoyl)ingenol	6.66
8	13.26	505.2980 337.1818	481.2945 [M-H]^-^	3-*O*-*(2′E*,*4′Z*-decadienoyl)-20-deoxyingenol	26.93
9	13.50	563.5580 503.2812 395.1912	585.3019 [M + HCOO]^-^	3-*O*-(*2*′*E*,*4*′*Z*-decadienoyl)-5-*O*-acetylingenol	95.25
10	14.61	563.3061 503.3406 395.1876 335.1636	539.0378 [M-H]^-^	3-*O*-(*2*′*E*,*4*′*Z*-decadienoyl)-20-*O*-acetylingenol	10.72
11	18.27	645.4619 [M + H]^+^ 627.4326 529.3592 511.3414 427.2519 329.1778 311.1680	689.4784 [M + HCOO]^-^	3-*O*-(2,3-dimethylbutanoyl)-13-*O*-dodecanoylingenol	17.40

For peak 11, a formate adduct molecular ion at *m/z* 689.4784 [M + HCOO]^-^ was observed in negative ion mode, molecular ion at *m/z* 645.4619 [M + H]^+^ and a series of fragment ions at *m/z* 627.432 [M + H-H_2_O]^+^, 529.3592 [M + H-C_6_H_12_O_2_]^+^, 511.3414 [M + H-C_6_H_12_O_2_-H_2_O]^+^, 427.2519 [M + H-C_12_H_24_O_2_-H_2_O]^+^, 329.1778 [M + H-C_6_H_12_O_2_- C_12_H_24_O_2_]^+^ and 311.1680 [M + H-C_6_H_12_O_2_-C_12_H_24_O_2_-H_2_O]^+^ were observed in positive ion mode, suggesting the presence of two long-chain groups. In agreement with the data in the literature [15, 16, 22], peak 11 was tentatively identified as 3-*O*-(2, 3-dimethylbutanoyl)- 13-*O*-dodecanoylingenol. It has cytotoxicity against human normal liver cell line L-O2 [14] and can enhance the replication of HIV-1 (human immunodeficiency virus type1) [23].

**Fig. 3:**
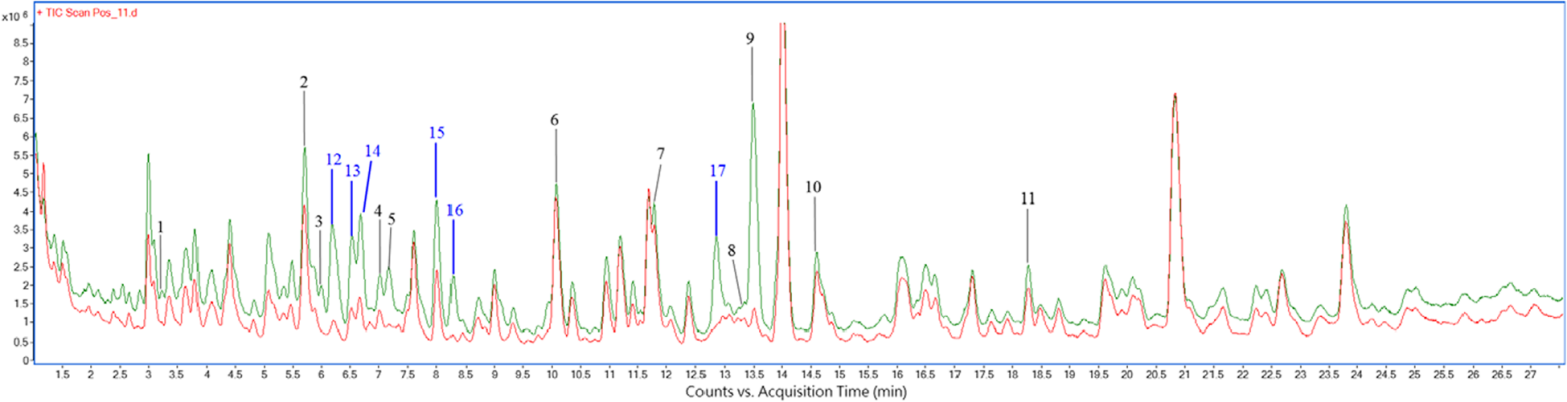
Overlapped representative total ion current chromatograms of *Gansui* samples in the positive-ion mode. The total ion current chromatograms of raw *Gansui* and vinegar-processed *Gansui* samples in the positive-ion mode were indicated by green line and red line, respectively

In addition to those 11 tentatively identified compounds, there were more changes (peaks 12-17) observed. An overlapped total ion current chromatograms of raw *Gansui* (indicated by green line) and vinegar-processed *Gansui* (indicated by red line) was shown in Fig. 3 with peaks 12-17 highlighted in blue color. Unfortunately, peaks 12-17 could not be identified according to the available data. Further work is required to assign them with potential identities. The results showed that the ion chromatograms prior to and after processing with vinegar actually varied, with several peaks significantly altered.

## Conclusions

In the present study, a comprehensive comparison of the chemical profiles between vinegar-processed and raw *Gansui* has been conducted using the UHPLC UHD Q-TOF MS/MS method. Results indicated that processing with vinegar caused conspicuous chemical changes in *Gansui*. Among the changed compounds, 11 toxic terpenoids, 3-*O*-benzoyl-13-*O*- dodecanoylingenol/20-*O*-benzoyl-13-*O*-dodecanoylingenol, kansuinine D, kansuinine A, 3-*O*-benzoyl-13-*O*-dodecanoylingenol/20-*O*-benzoyl-13-*O*-dodecanoylingenol, 3-*O*-benzoylingenol/20-*O*-benzoylingenol, 20-*O*-(*2*′*E*,*4*′*Z*-decadienoyl)ingenol/20-*O*-(*2′E*,*4′E*- decadienoyl)ingenol/3-*O*-(*2′E*,*4′Z*-decadienoyl)ingenol/3-*O*-(*2*′*E*,*4*′*E*-decadienoyl)ingenol, 3-*O*-*(2′E*,*4′Z*-decadienoyl)-20-deoxyingenol,3-*O*-(*2*′*E*,*4*′*Z*-decadienoyl)-5-*O*-acetylingenol,3-*O*-(*2*′*E*,*4*′*Z*-decadienoyl)-20-*O*-acetylingenol,3-*O*-(2,3-dimethylbutanoyl)-13-*O*-dodecanoylingenol, were tentatively identified. The contents of most of these terpenoids were significantly decreased after processing with reductions of 6.66-95.25 %. These findings can help us understand the chemical basis for the toxicity reduction of *Gansui* afforded by processing with vinegar. To establish the relationship between processing-induced chemical changes and the reduction of toxicity of *Gansui*, further investigations are warranted.

## References

[1] Miyata S, Wang LY, Yoshida C, Kitanaka S (2006). Inhibition of cellular proliferation by diterpenes, topoisomerase II inhibitor. Bioorgan Med Chem.

[2] Wang LY, Wang NL, Yao XS, Miyata S, Kitanaka S (2002). Diterpenes from the roots of *Euphorbia kansui* and their in vitro effects on the cell division of Xenopus. J Nat Prod.

[3] Wang LY, Wang NL, Yao XS, Miyata S, Kitanaka S (2003). Diterpenes from the roots of *Euphorbia kansui* and their in vitro effects on the cell division of Xenopus (part 2). Chem Pharm Bull.

[4] Wu TS, Lin YM, Haruna M, Pan DJ, Shingu T, Chen YP (1991). Antitumor agents, 119. Kansuiphorins A and B, two novel antileukemic diterpene esters from *Euphorbia kansui*. J Nat Prod.

[5] Yu FR, Lian XZ, Guo HY, McGuire PM, Li RD, Wang R (2005). Isolation and characterization of methyl esters and derivatives from *Euphorbia kansui* (Euphorbiaceae) and their inhibitory effects on the human SGC-7901 cells. J Pharm Pharm Sci.

[6] Zheng WF, Cui Z, Zhu Q (1998). Cytotoxicity and antiviral activity of the compounds from *Euphorbia kansui*. Planta Med.

[7] Blanco-Molina M, Tron GC, Macho A, Lucena C, Calzado MA, Munoz E (2001). Ingenol esters induce apoptosis in jurkat cells through an AP-1 and NF-κB independent pathway. Chem Biol.

[8] Nunomura S, Kitanaka S, Ra C (2006). 3-O-(2,3-dimethylbutanoyl)-13-O- decanoylingenol from *Euphorbia kansui* suppresses IgE-mediated mast cell activation. Biol Pharm Bull.

[9] Pan Q, Ip FC, Ip NY, Zhu HX, Min ZD (2004). Activity of macrocyclic jatrophane diterpenes from *Euphorbia kansui* in a TrkA fibroblast survival assay. J Nat Prod.

[10] Matsumoto T, Cyong JC, Yamada H (1992). Stimulatory effects of ingenols from *Euphorbia kansui* on the expression of macrophage Fc receptor. Planta Med.

[11] Pan Q, Da Min Z (2002). Studies on the structure of kansuinine A from *Euphorbia kansui*. Chin Chem Lett.

[12] Hirata Y (1975). Toxic substances of Euphorbiaceae. Pure Appl Chem.

[13] Committee NP (2010). Pharmacopoeia of the People’s Republic of China. Part.

[14] Yan X, Zhang L, Guo J, Cao Y, Shang E, Tang Y (2014). Processing of kansui roots stir-baked with vinegar reduces kansui-induced hepatocyte cytotoxicity by decreasing the contents of toxic terpenoids and regulating the cell apoptosis pathway. Molecules.

[15] Zhang L, Shu X, Ding A, Yu L, Tang Y, Duan J (2009). LC–DAD–ESI-MS–MS separation and chemical characterization of the inflammatory fraction of the roots of *Euphorbia kansui*. Chromatographia.

[16] Liu Y, Liu ZQ, Li HL, SONG FR, LIU SY (2008). Studies on diterpenoids constituents from Euphorbia kansui by electro-spray ionization multi-stage tandem mass spectrometry. Chem J Chinese U.

[17] Wang H, Wang J, Luo J, Kong L (2013). Isolation of ingenol-type diterpenoids from *Euphorbia kansui* by offline coupling of HPLC-ESI-MSn and HSCCC. Sep Sci Technol.

[18] Zhang L, Gao L, Li Z, Yan X, Yang Y, Tang Y (2012). Bio-guided isolation of the cytotoxic terpenoids from the roots of *Euphorbia kansui* against human normal cell lines L-O2 and GES-1. Int J Mol Sci.

[19] Halaweish FT, Kronberg S, Hubert MB, Rice JA (2002). Toxic and aversive diterpenes of *Euphorbia esula*. J Chem Ecol.

[20] Wang HY, Wang JS, Wei DD, Wang XB, Luo J, Yang MH (2012). Bioactivity-guided isolation of antiproliferative diterpenoids from *Euphorbia kansui*. Phytother Res.

[21] Shen J, Mo X, Tang Y, Zhang L, Pang H, Qian Y (2013). Analysis of herb-herb interaction when decocting together by using ultra-high-performance liquid chromatography-tandem mass spectrometry and fuzzy chemical identification strategy with poly-proportion design. J Chromatogr A.

[22] Wang YB, Li YY, Wang HB, Qin GW (2007). Chemical constituents from the roots of *Euphorbia kansui*. Chin J Nat Med.

[23] Fujiwara M, Okamoto M, Ijichi K, Tokuhisa K, Hanasaki Y, Katsuura K (1998). Upregulation of HIV-1 replication in chronically infected cells by ingenol derivatives. Arch Virol.

[24] Pan DJ, Hu C, Chang JJ, Thomas TYL, Chen YP, Hsu HY (1991). Kansuiphorin C and D, cytotoxic diterpenes from *Euphorbia kansui*. Phytochemistry.

[25] Appendino G, Tron GC, Cravotto G, Palmisano G, Annunziata R, Baj G (1999). Synthesis of modified ingenol esters. Eur J Org Chem.

